# Cardiopulmonary responses to acute exogenous ketosis at rest, and during submaximal and maximal exercise

**DOI:** 10.14814/phy2.70397

**Published:** 2025-05-29

**Authors:** Christopher D. Crabtree, Justen Stoner, Alex Buga, Bradley Robinson, Drew Decker, Ashwini Chebbi, Xavier El‐Shazly, Emily Murphy, Aydan Jordan, Teryn Bedell, Madison L. Kackley, Tyler McClure, Yuchi Han, Orlando P. Simonetti, Jeff S. Volek

**Affiliations:** ^1^ Department of Radiology The Ohio State University Columbus Ohio USA; ^2^ Department of Human Sciences The Ohio State University Columbus Ohio USA; ^3^ Division of Cardiovascular Medicine, Department of Internal Medicine The Ohio State University Columbus Ohio USA

**Keywords:** cardiopulmonary, exercise performance, ketones, ketosis

## Abstract

Nutritional ketosis achieved through various methods in animals and humans has been shown to augment cardiac efficiency and function. However, this response during exercise has not been well characterized. Recreationally active adults (*n* = 12) completed a double blind, balanced, placebo‐controlled, crossover study to examine the effects of bis‐octanonyl (*R*)‐1,3‐butanediol (BO‐BD) ingestion on cardiopulmonary function at rest and during a maximal oxygen consumption (V̇O_2max_) treadmill test (Bruce Protocol). Participants presented to the testing facility fasted. Capillary blood samples were obtained to measure glucose and beta‐hydroxybutyrate (*R*‐βHB) prior to consuming the BO‐BD or a calorically matched placebo (PL) beverage. Metabolic and cardiovascular measures were collected every 15–30 min following beverage consumption. Participants began the V̇O_2max_ test 120 min post‐beverage ingestion. At rest, capillary *R*‐βHB elevated rapidly after BO‐BD ingestion and continued to steadily increase to 2.4 mM prior to the maximal exercise test. During the 120 min rest period, BO‐BD increased resting heart rate (HR) (*p* = 0.001), ventilation (*p* < 0.001), and V̇O_2_ (*p* = 0.002) relative to PL. Although the total time to exhaustion was similar between conditions, V̇O_2max_ was lower after BO‐BD (*p* < 0.001). There were no differences in exercise lactate, RER, respiration, or rating of perceived exertion (RPE) between conditions. Compared to PL, BO‐BD rapidly achieves nutritional ketosis, increases resting cardio‐respiratory parameters, but somewhat paradoxically decreases peak aerobic exercise oxygen consumption despite achieving similar peak workloads.

## 1 INTRODUCTION

Nutritional ketosis is operationally defined as having a circulating *R*‐βHB concentration between 0.5 and 5.0 mM (Volek et al., 2024). A state of nutritional ketosis supplies a readily usable and efficient oxidative metabolic substrate to organs such as the brain, heart, and skeletal muscle (Laffel, 1999). Sodium‐glucose cotransporter 2 inhibitors (SGLT2i) cause a modest rise in circulating ketone levels into the range of nutritional ketosis (Ferrannini, Baldi, et al., 2016; Polidori et al., 2018). In patients with heart failure (HF), SGLT2i have been overwhelmingly successful in improving patient outcomes and mortality (Verma & McMurray, 2018; Zinman et al., 2015). These observations have in part contributed to a reexamination of the potential favorable role of ketones on cardiovascular function (Mudaliar et al., 2016; Yurista et al., 2021, 2022). Various acute advanced imaging studies have shown exogenous ketones to confer a rapid increase in cardiac output in a ketone dose‐dependent fashion (Nielsen et al., 2019) in both healthy adults (Gormsen et al., 2017; Nielsen et al., 2019; Selvaraj et al., 2022) and patients with various chronic cardiopulmonary diseases (Berg‐Hansen et al., 2023; Nielsen et al., 2023) including heart failure (Nielsen et al., 2019). While these acute ketone investigations observed improved cardiovascular function and mechanics in the resting state, the response during exercise has not been well characterized. Further investigation is necessary for translation to patient populations with impaired functional capacity, such as in HF.

Numerous studies have observed that ketogenic diets (KDs) enhance reliance on lipid metabolism (Harvey et al., 2019) in chronic feeding interventions (Shaw, Merien, Braakhuis, Maunder, & Dulson, 2019) as well as in elite athletes habitually consuming KD (Volek et al., 2016). Enhanced fat utilization during rest and submaximal exercise (Shaw, Merien, Braakhuis, Maunder, & Dulson, 2019; Volek et al., 2016) can improve the metabolic flexibility of the heart, increasing its adaptability to changing exercise intensity and stimuli (Smith et al., 2018). However, there have also been studies showing negligible improvement (Carr et al., 2018; Cipryan et al., 2018; Shaw, Merien, Braakhuis, Maunder, & Dulson, 2019) or reduced aerobic performance following a KD intervention (Burke et al., 2017).

Nutritional ketosis can also be induced acutely using exogenous ketones, such as with ketone esters (KE) or with ketogenic promoting beverages formulated with ketone precursors, such as Bis‐Octanoyl (*R*)‐1,3‐butanediol (BO‐BD) (Stubbs et al., 2023); both commercially available in powder or liquid beverage form. Compared to consuming pro‐ketones, ingestion of BO‐BD mimics endogenous ketogenesis by providing ketogenic precursors (i.e., two, 8‐carbon medium‐chain fatty acids esterified to (*R*)‐1,3‐butanediol) that are readily converted to *R*‐βHB by the liver. This results in a dose‐dependent plasma *R*‐βHB elevation over several hours (Crabtree et al., 2023; Stubbs et al., 2017) without requiring dietary modifications or long‐term adherence. Exogenous ketone consumption has been associated with improved exercise economy (i.e. requiring less oxygen consumption for the same workload) and lactate response, indicative of altered oxygen and metabolic substrate dynamics during exercise (Brady & Egan, 2024; Da Costa et al., 2020; Evans & Egan, 2018).

Investigation of cardiometabolic performance under conditions of exercise stress following ketone supplementation is needed to understand the potential effects of these supplements in patient populations with reduced cardiovascular function, cardiac reserve, and exercise capacity. In these populations, the impact of increased circulating BHB across conditions of rest and stress is clinically relevant for patient safety (Yurista et al., 2021, 2022) to ensure enhanced resting blood flow (Nielsen et al., 2019) does not compromise cardiac reserve and thus oxygen availability during stress. The limited research combined with their mixed results collectively indicate that more work is needed to fully understand the acute effects of exogenous ketone supplementation on the cardiorespiratory response to exercise (i.e., heart rate, ventilation, oxygen consumption etc.) and exercise performance.

In this study, a group of healthy, recreationally active adults consumed a ketogenic‐promoting drink containing BO‐BD or a placebo (PL) on different test days and underwent a graded treadmill exercise evaluation to examine differences in metabolic and cardiorespiratory responses at rest and during exercise. We hypothesized that consumption of BO‐BD would enhance resting cardiorespiratory function and aerobic exercise performance compared to a placebo.

## 2 METHODS

**FIGURE 1:**
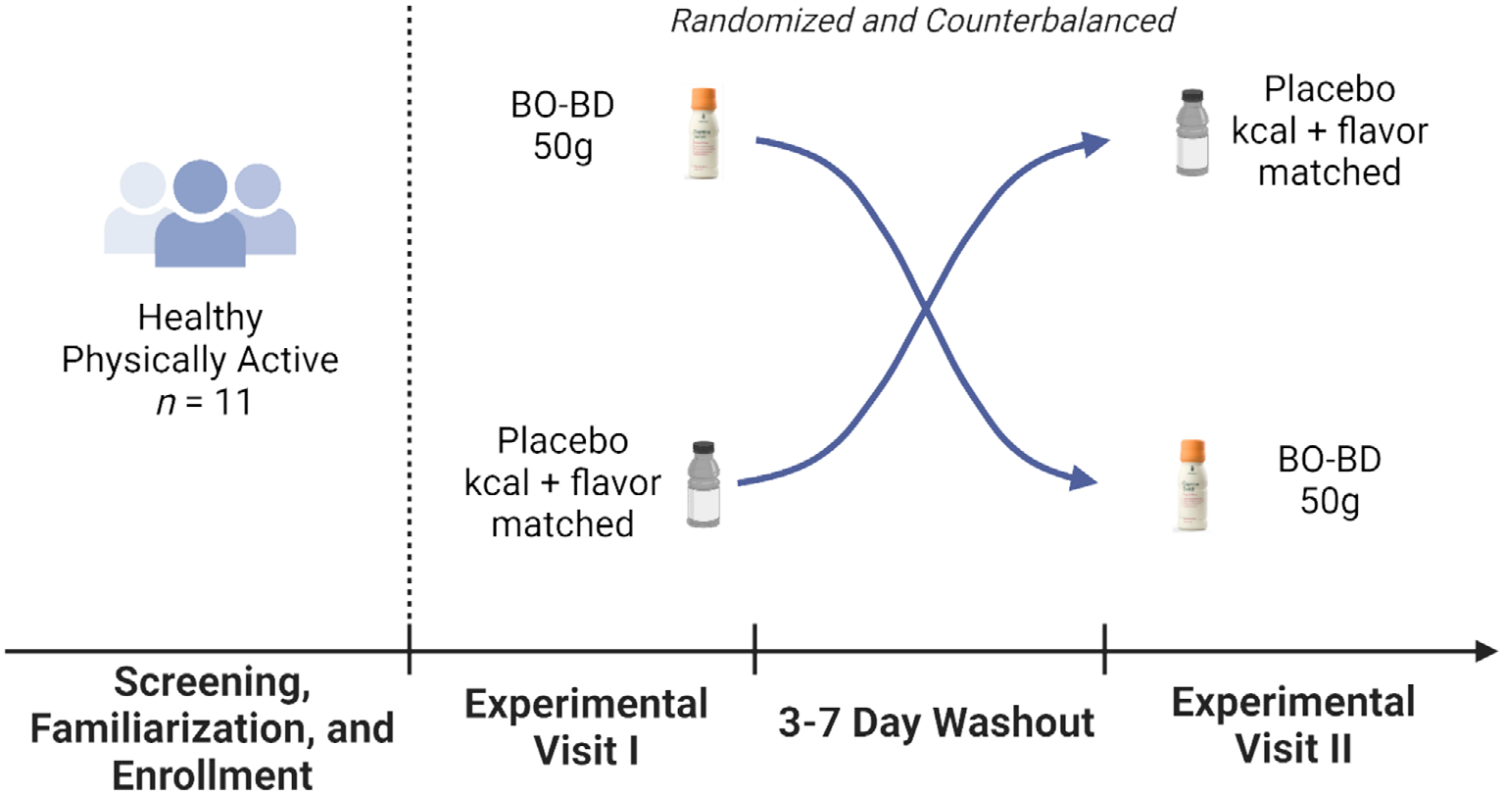
Study Visit Timeline. Healthy, physically active adults were randomized and counterbalanced to consume 50 g BO‐BD or calorie‐and flavor‐matched placebo. All participants were crossed over to consume the other supplement following a three‐ to seven‐day washout period.

This study was approved by the Institutional Review Board at the Ohio State University, and all participants provided written informed consent prior to participation (2022H0341). This randomized, placebo‐controlled, double‐blind, balanced cross‐over study analyzed the effects of acute exogenous BO‐BD ingestion on metabolic, cardiorespiratory, and exercise performance responses during a V̇O_2_ max test. This study was conducted in physically active healthy adults (age 18–65 years old) as determined by responses to exercise surveys (International Physical Activity Questionnaire) administered during the consent visit (Craig et al., 2003). During the consent visit, participants underwent a light familiarization protocol meant purely to acclimate subjects to the exercise protocol. During light familiarization, participants underwent a Bruce Protocol demonstration, performing the first ~30 s of each of the first few stages to acclimate themselves to the changing speed and incline inherent to the exercise protocol without inducing fatigue. Participants were non‐obese (BMI 18–30 kg/m^2^) and had no allergies to the ingredients in either of the study beverages. Exclusion criteria included current habitual consumption of a ketogenic or other low carbohydrate diets, pregnancy, and history of hypertension, smoking, or alcoholism. Study design scheme is described in Figure 1.

Participants arrived at the testing facility following an overnight fast (10‐12 h), refraining from caffeine for 24 h, and well hydrated. Hydration status was measured via specific gravity (USG); if dehydrated (USG >1.020), study staff offered participants 500 mL of water and retested their urine hydration on a subsequent void. Height and weight were measured to the nearest 0.1 cm and kg, respectively, using an electronic stadiometer (SECA 703 Digital, Hamburg, Germany). Other baseline measures included blood pressure (BP) systolic and diastolic blood pressure (SBP/DBP), continuous heart rate monitoring using a chest strap (Polar H10, Polar Electro OY, Kempele, Finland), and capillary ketone and glucose levels using a portable reagent strip device (Keto Mojo, Keto‐Check Inc., Savannah, GA). Following the collection of all baseline measures, the subject ingested the test product (BO‐BD or PL) in a blinded and randomized manner. Capillary *R*‐βHB/glucose, blood pressure, and heart rate were measured at 15, 30, 60, and 90 min following consumption, and again immediately before starting the maximal exercise test.

Respiratory gases were collected via open spirometry and analyzed using a metabolic measurement system (Parvomedic TrueOne 2400). Ventilatory gases were collected between 0 and 10 min, 60 and 70 min, and 110 and 120 min following consumption during the resting phase to measure oxygen consumption (V̇O_2_), ventilation (V̇E), tidal volume (V̇T), respiratory rate (RR), the volume of carbon dioxide production (V̇CO_2_), and ventilatory efficiency (V̇E/V̇CO_2_). All respiratory measures were collected during the last 5 min of the 10‐min gas collection phase. At 120 min following consumption, the participants began the maximal exercise test. The recovery phase began following exercise cessation, including additional capillary blood sampling 5 min post exercise.

### 2.1 Maximal oxygen consumption test – Bruce protocol

The Bruce Protocol is a graded exercise treadmill test consisting of 3‐min stages with standardized increments in speed and elevation to increase exercise intensity (Fletcher et al., 2013). Maximal treadmill graded exercise is a safe and reliable measure designed to assess maximal oxygen consumption (V̇O_2_max) in healthy adults. The exercise protocol ended when the participant reached volitional failure (i.e., quit at will). V̇O_2_max criteria consisted of obtaining at least two of the following three criteria: RER ≥1.10, postexercise lactate levels ≥8.0 mM, and RPE ≥17 (Balady et al., 2010; Liguori & Medicine (ACSM) AC of S, 2020). Various measures, such as HR, BP, ratings of perceived exertion (RPE) (Borg, 1982), and capillary blood lactate, were collected throughout the first three stages of exercise to monitor progress and safety. Lactate and BP were not collected beyond the first three stages to avoid impacting exercise performance. Gases were collected throughout the entire exercise test (Parvomedic TrueOne 2400 metabolic cart) to calculate V̇E, V̇T, RR, V̇CO_2_, and V̇E/V̇CO_2_, which were collected and averaged during the entire 3 min of each stage, while V̇O_2_ was recorded as the peak oxygen consumption rate from each stage. All exercise sessions were monitored throughout by a team of trained exercise physiologists to ensure subject safety and to provide motivation.

### 2.2 Beverage description

This study featured two test articles: BO‐BD and a placebo. BO‐BD is a commercially available novel ketone diester, bis‐octanoyl (*R*)‐1,3‐butanediol, sold in liquid beverage and powder forms (Stubbs et al., 2023). Two servings of the liquid beverage were consumed together (50 g total) to achieve BHB concentrations shown to rapidly elevate cardiac output (Gormsen et al., 2017; Nielsen et al., 2019). The BO‐BD serving had a macronutrient composition of 4 g of carbohydrate, 1 g of fat, 4 g of protein, and 50 g of bis‐octanoyl (*R*)‐1,3‐butanediol and contained water, C8 Ketone Di‐ester, High Fat Whey Protein Concentrate, Modified Gym Acacia, Citric Acid, Soy Lecithin, Natural Flavors, Stevia Leaf Extract, Pectin, Sodium Carboxymethyl Cellulose, and Potassium Sorbate. A fat‐based placebo was formulated with standard dietary ingredients including water, high fat whey protein concentrate, canola oil, and artificial sweeteners, matched to the BO‐BD for volume and caloric content. The macronutrient breakdown for the placebo product included 4 g of carbohydrate, 50 g of fat, and 4 g of protein. Both test articles were administered in an opaque bottle to maintain the double‐blinded manner of the protocol.

### 2.3 Statistical analysis

Statistics were performed using commercially available statistics software (SPSS IBM Version 28.0. Armonk, NY: IBM Corp). A priori two‐tail *α* significance was set at 0.05. Main effects and interactions were analyzed using a repeated measures analysis of variance (ANOVA). Significant effects and interactions were further inspected with Bonferroni‐corrected post‐hoc tests or appropriate non‐parametric statistical tests. Resting measures of ketones, glucose, lactate, HR, RER, V̇E, V̇T, V̇CO_2_, and V̇O_2_ values over time were analyzed using a 2 (condition) by 6 (time) repeated measures ANOVA. V̇O_2_max and total exercise duration were compared at peak exercise between placebo and BO‐BD using paired *t*‐tests. Staged analysis of exercise results was constrained to the highest workload stage that all participants completed along with maximal results as previously described (Nicolò et al., 2019). Exercise HR, RER, V̇E, V̇T, V̇O_2_, and were inspected using a 2 × 3 repeated measures ANOVA. Estimated marginal means were employed using a linear mixed‐effects model to generate unbiased lactate without exposing the dataset to listwise deletion and loss of statistical power values where data was missing at random due to equipment failure.

## 3 RESULTS

### 3.1 Demographics

All participants (*n* = 12) completed all study protocols with no differences in body anthropometrics between test visits (*p* > 0.05). One subject was dropped from data reporting due to self‐reported illness onset that prompted a delay of the second test visit. Thus, the full analyzed cohort was *n* = 11. Some data collected during exercise was lost; in this case, data reporting will mention the sample size only if it is different from the full *n* = 11 cohort. Baseline body anthropometrics, capillary R‐BHB, glucose, and lactate were all similar at baseline between test visits (*p* > 0.05) (Table 1).

**TABLE 1:** Participant Characteristics.

Variable	Test visit 1	Test visit 2
Sex	1F: 10M	
Age (yrs)	27.08 ± 8.8	
Height (cm)	173 ± 6.5	
Weight (kg)	76.4 ± 6.6	76.5 ± 6.7
BMI (kg/m^2^)	25.6 ± 2.0	25.6 ± 2.1

*Note*: Values are Mean ± SD.

### 3.2 Resting responses

**FIGURE 2:**
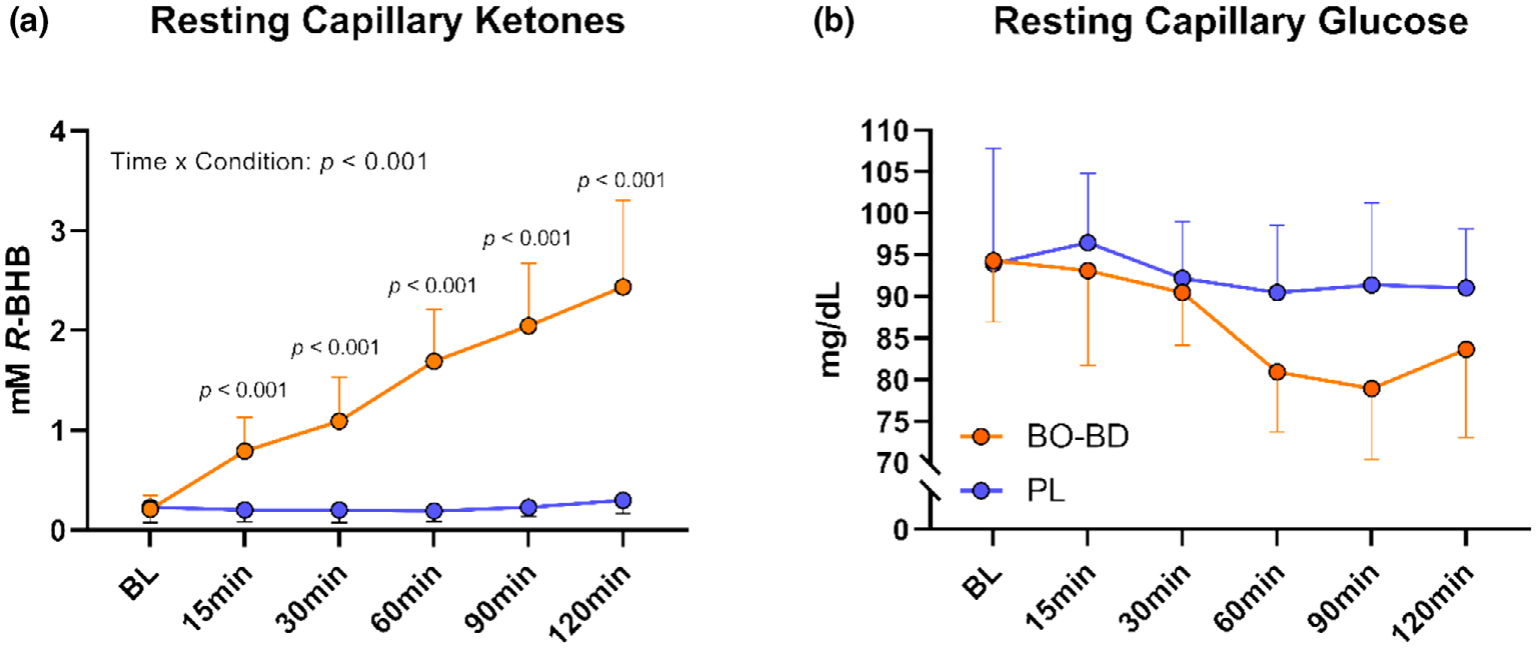
Metabolic response at rest following supplementation. (a) Kinetic ketone curves throughout the resting phase following supplementation and (b) Kinetic glucose curves throughout the resting phase following supplementation. All p values represent between condition effects occuring within a given timepoint.

Following consumption of the BO‐BD, R‐BHB increased gradually throughout the 120 min post‐consumption resting phase (*p* < 0.001) and differed relative to PL, which did not affect R‐BHB concentration (*p* < 0.001), resulting in a significant interaction effect (*p* < 0.001) (Figure 2a). R‐BHB was highest at 120 min with a mean of 2.4 mM (range 1.0–4.3 mM). Glucose decreased (*p* < 0.001) following ingestion of either beverage, with a greater effect after BO‐BD (*p* = 0.017), producing a trend for a significant interaction effect of condition*time (*p* = 0.06) (Figure 2b). Capillary lactate did not differ between groups nor change during the resting timeline (*p* > 0.05).

**FIGURE 3:**
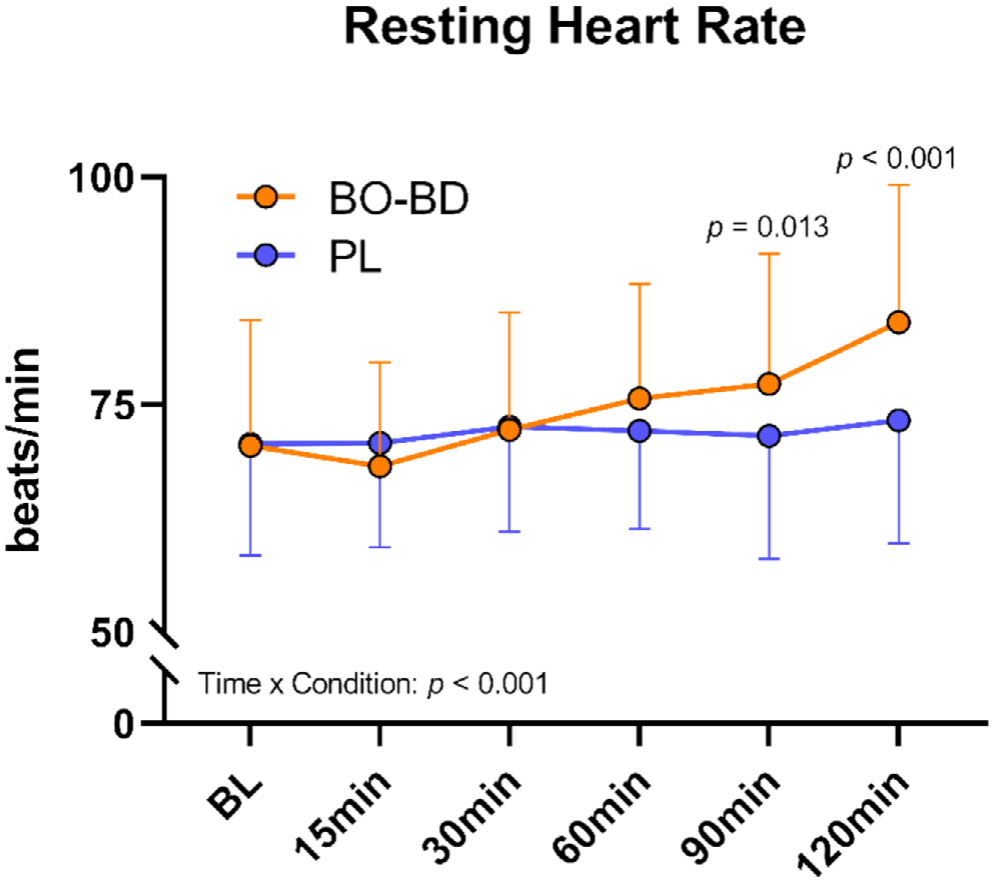
Heart rate response during resting phase following supplementation. All p values represent between condition effects occuring within a given timepoint.

HR increased following beverage ingestion at rest (*p* = 0.004) but was similar between conditions (*p* = 0.21). However, there was a significant interaction effect (*p* < 0.001) reflecting a delayed elevation in HR post‐BO‐BD consumption at 120 min just prior to exercise (Figure 3). Resting systolic and diastolic blood pressure (SBP and DBP, respectively) did not change after either beverage (*p* > 0.05).

Respiratory exchange ratio (RER) decreased (*p* = 0.003) during the resting phase of the study similarly between conditions (*p* = 0.13). This produced an interaction effect (*p* = 0.043) in which post‐hoc analysis revealed BO‐BD to confer elevated RER (0.80 ± 0.03) compared to PL (0.76 ± 0.05) at the 120 min timepoint (*p* = 0.04) immediately before exercise.

Resting oxygen consumption (V̇O_2_) rose steadily throughout the resting phase of the study (*p* = 0.003) in the BO‐BD condition while the PL condition remained near baseline (*p* = 0.006). This resulted in a significant interaction effect (*p* = 0.003). Post‐hoc analysis revealed greater resting oxygen consumption at 60 min (*p* = 0.01) and 120 min (*p* = 0.018) following BO‐BD consumption compared to the PL.

**FIGURE 4:**
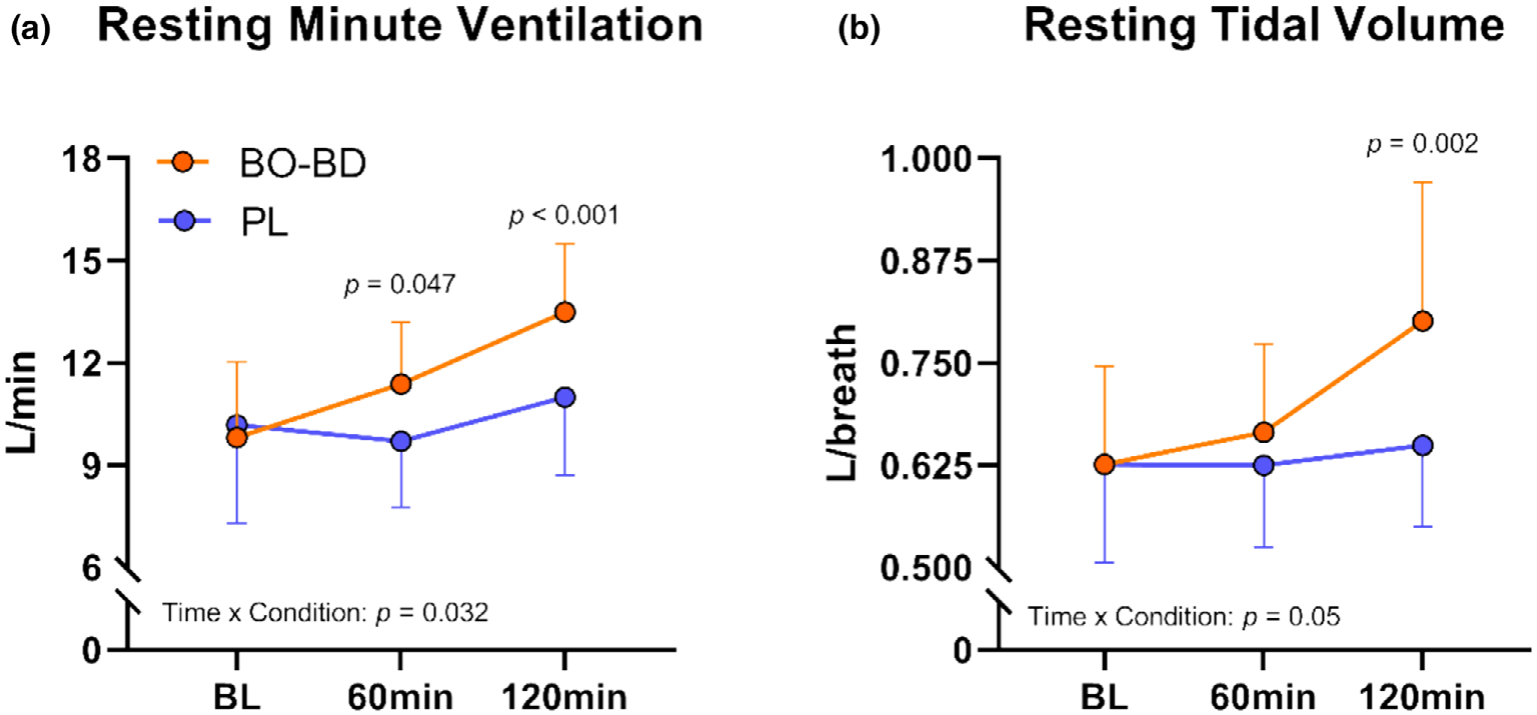
Respiratory response at rest following supplement consumption. (a) Ventilation response at rest following supplement consumption. (b) Tidal volume response at rest following supplement consumption. All p values represent between condition effects occuring within a given timepoint.

An interaction effect (*p* = 0.032) for resting minute ventilation (V̇E) was observed as V̇E measurements increased from baseline to immediately before exercise in the BO‐BD condition (*p* < 0.001), while ventilation remained near baseline levels in the PL condition producing differential supplement responses (*p* = 0.016) (Figure 4a). Similarly, an interaction effect (*p* = 0.05) was observed in tidal volume as resting values increased following BO‐BD consumption (*p* = 0.03), while values remained unchanged following PL consumption, resulting in a supplement effect (*p* < 0.001) (Figure 4b). No differences were observed in respiratory rate over time (*p* = 0.356), between conditions (*p* = 0.289), or the interaction of time*condition (*p* = 0.072).

**FIGURE 5:**
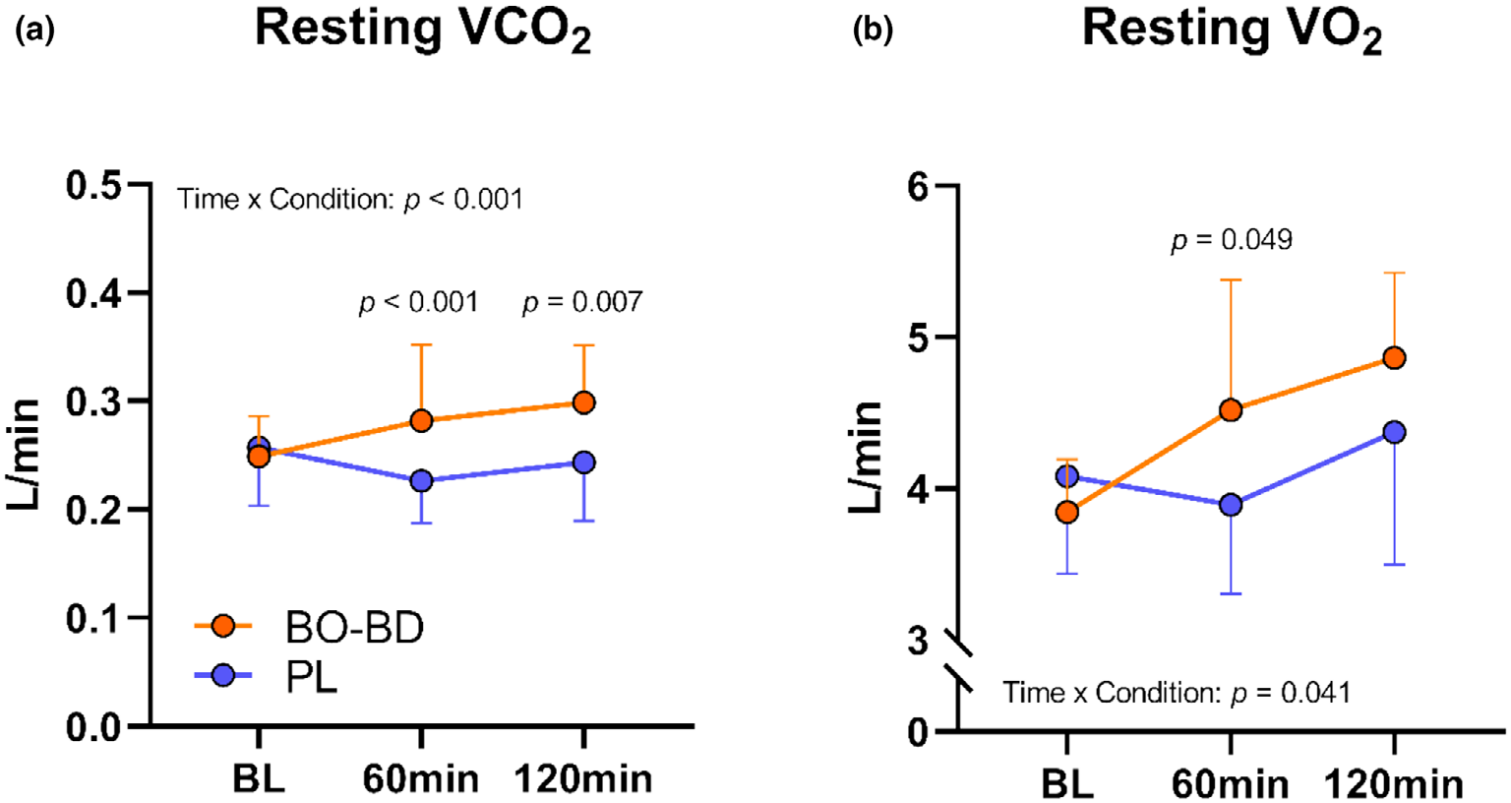
Respiratory V̇CO_2_ response following supplement consumption. All p values represent between condition effects occuring within a given timepoint.

An interaction effect of time*condition (*p* < 0.001) was observed for resting V̇CO_2_ as the V̇CO_2_ response differed following supplementation (*p* = 0.003) (Figure 5a). There were significant main effects of time (*p* = 0.003) and condition (*p* = 0.014) with an interaction effect of time*condition (*p* = 0.041) being observed for resting V̇O_2_. Post‐hoc analysis revealed significantly different responses at 60 min post‐consumption (*p* = 0.049) as BO‐BD consumption resulted in increases in resting oxygen consumption compared to PL consumption (Figure 5b). Ventilatory efficiency (V̇E/V̇CO_2_) increased throughout the duration of the resting period regardless of condition (*p* = 0.014), but no condition differences or interaction effects were observed during the resting phase.

### 3.3 Exercise responses

**FIGURE 6:**
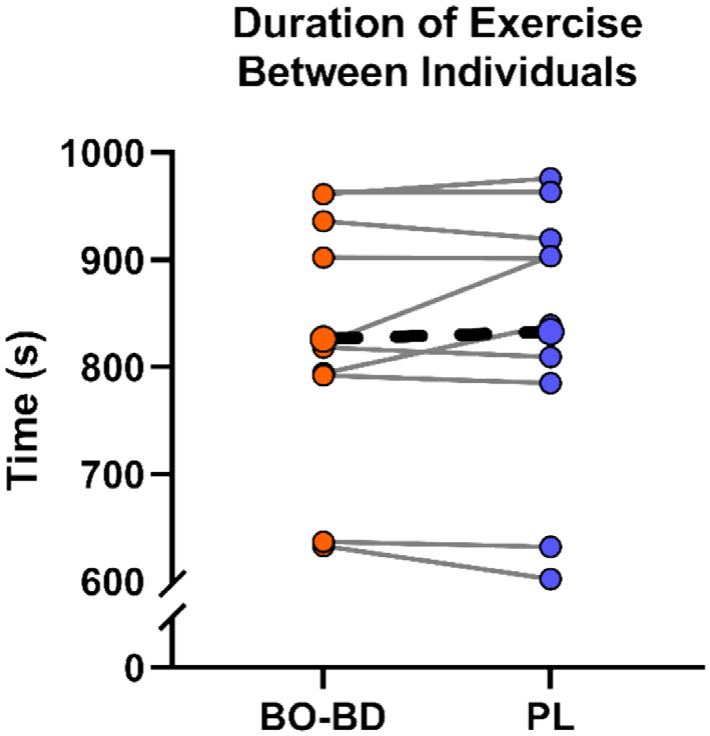
Individual duration of Bruce Protocol (solid lines) between supplement conditions (*n* = 10) compared to the group averages (dotted line).

All participants completed at least the first three stages of the exercise protocol. There was no difference (*p* = 0.51) in total exercise duration between BO‐BD (825.8 ± 120.0 s) and PL (832.9 ± 129.0 s) (*n* = 10) (Figure 6). RPE increased throughout exercise duration (*p* < 0.001) but did not differ between BO‐BD (19.4 ± 1.0) or PL (19.3 ± 1.2) (*p* = 0.54). RER increased throughout exercise (*p* < 0.001) to similar peaks between BO‐BD (1.11 ± 0.08) and PL (1.11 ± 0.08) and decreased following exercise cessation regardless of condition (*p* = 0.42). All participants achieved at least two of the V̇O_2_ max criteria with 19/20 postexercise lactate levels (13.9 ± 3.4), 20/20 reported RPE values (19.3 ± 1.1), and 13/20 peak RER values (1.11 ± 0.07) of analyzed exercise tests.

**FIGURE 7:**
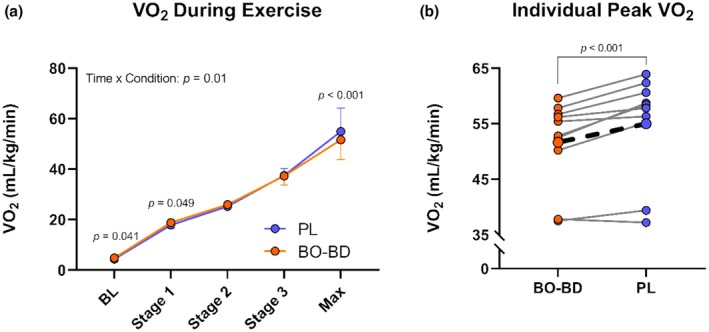
(a) V̇O_2_ per stage compared between supplement conditions (*n* = 10). (b) Individual peak V̇O_2_ (solid lines) between supplement conditions compared to the group averages (dotted line). *p ≤ 0.05, ***p ≤ 0.001 versus PL within a given time point.

V̇O_2_ increased throughout exercise (*p* < 0.001) and was similar between conditions (*p* = 0.32) yet produced a significant interaction effect (*p* = 0.01) (Figure 7a) (*n* = 10). Despite similar exercise duration between conditions, oxygen consumption at peak exercise was reduced 6% following BO‐BD (51.7 ± 7.9 mL/min/kg) compared to PL (55.0 ± 9.2 mL/min/kg) (*p* ≤ 0.001). This was relatively consistent between participants, as only 1/10 participants reached a slightly higher V̇O_2_ max following BO‐BD consumption, and that subject had the lowest V̇O_2_max (Figure 7b).

All cardiorespiratory exercise measures are listed in Table 2 for each stage of exercise. All metrics of cardiorespiratory performance increased throughout exercise duration and intensity (*p* < 0.001). All cardiopulmonary differences between supplements at rest (HR, V̇E, V̇T, and V̇CO_2_) maintained throughout submaximal exercise before converging at maximal exercise (*p* > 0.05).

**TABLE 2:** Cardiorespiratory exercise response.

Variable	Supplement	Pre‐exercise	Stage 1	Stage 2	Stage 3	Exercise max	2 × 5 RM ANOVA (*p*‐value)		
							Supp.	Time	Interaction
Heart Rate (bpm)	BO‐BD	87.9 ± 14.8*	120.1 ± 16.1*	141.5 ± 16.4*	165.6 ± 14.4	188.9 ± 8.9	0.020^*^	<0.001^***^	0.013^***^
	PL	74.1 ± 16.9	109.3 ± 16.9	132.8 ± 20.6	160.3 ± 15.1	188.5 ± 12.7			
Ventilation (L/min)	BO‐BD	13.5 ± 2.0*	26.0 ± 3.9*	39.3 ± 5.3*	61.6 ± 7.7*	127.3 ± 20.7	0.067	<0.001^***^	0.015^**^
	PL	10.8 ± 2.3	23.6 ± 3.3	36.3 ± 4.8	58.8 ± 8.3	129.6 ± 22.7			
Tidal Volume (L)	BO‐BD	0.80 ± 0.17**	1.30 ± 0.15**	1.86 ± 0.18**	2.35 ± 0.31*	2.84 ± 0.40	<0.001^***^	<0.001^***^	<0.001^***^
	PL	0.64 ± 0.10	1.14 ± 0.14	1.70 ± 0.17	2.26 ± 0.33	2.90 ± 0.42			
RR (breaths/min)	BO‐BD	18.3 ± 2.3	20.1 ± 2.6	21.3 ± 3.2	26.7 ± 5.0	45.1 ± 6.7	0.990	<0.001^***^	0.603
	PL	17.0 ± 2.0	20.9 ± 2.4	21.5 ± 3.0	26.5 ± 4.7	44.7 ± 7.9			
V̇CO_2_ (L/min)	BO‐BD	0.023 ± 0.004*	0.054 ± 0.008*	0.093 ± 0.009*	0.152 ± 0.017	0.322 ± 0.055	0.46	<0.001^***^	0.035^*^
	PL	0.018 ± 0.004	0.050 ± 0.008	0.089 ± 0.010	0.153 ± 0.018	0.339 ± 0.058			
V̇O_2_ (L/min)	BO‐BD	0.37 ± 0.04*	1.44 ± 0.11*	1.98 ± 0.10	2.85 ± 0.28	3.95 ± 0.60***	<0.001^***^	0.32	<0.001^***^
	PL	0.33 ± 0.07	1.36 ± 0.13	1.93 ± 0.13	2.87 ± 0.22	4.2 ± 0.70			

*Note*: Values reported as mean ± SD. *p ≤ 0.05, **p ≤ 0.01, ***p ≤ 0.001. All * represent between group differences within a given exercise stage.

Abbreviations: RM ANOVA, repeated measures analysis of variance; RR, respiratory rate; V̇CO_2_, volume of carbon dioxide production; V̇O_2_, volume of oxygen consumption.

SBP rose throughout exercise duration (*p* < 0.001) similarly between supplement conditions (*p* = 0.22). DBP remained stable throughout exercise regardless of supplement consumed (*p* > 0.05). Mean arterial pressure (MAP) increased (*p* < 0.001) during exercise influenced by changes in SBP, but was not different between groups (*p* = 0.23).

Lactate increased steadily throughout the exercise protocol (*p* < 0.001) regardless of condition (*p* = 0.94). Compared to immediately before exercise onset, R‐BHB decreased (*p* < 0.001) in the BO‐BD group (*p* < 0.001) while the PL group remained near baseline; glucose, meanwhile, increased following exercise cessation compared to immediately before exercise onset (*p* < 0.001) independent of the supplement consumed (*p* = 0.25).

## 4 DISCUSSION

A cohort of twelve healthy and physically active adults consumed two different supplements, a ketogenic promoting beverage containing 50 g of BO‐BD, and a calorie/volume‐matched fat‐based placebo, to investigate the effects of acute nutritional ketosis on metabolic, cardiorespiratory, and exercise performance responses prior to and during a maximal oxygen consumption test. At rest, BO‐BD consumption increased multiple aspects of cardiopulmonary and ventilatory function, lowered blood glucose, and led to a rapid elevation of blood R‐BHB that persisted throughout rest, submaximal, and maximal exercise. Despite achieving a similar total exercise duration and exercise workload between supplement conditions, peak oxygen consumption was consistently lower, resulting in a mean 6% decrease following BO‐BD ingestion, suggesting a ketone‐mediated differential change in oxygen consumption that manifested at rest (increased) and peak V̇O_2_ max (decreased).

BO‐BD consumption gradually increased blood *R*‐βHB availability over the 2 h resting period prior to exercise. This has been observed previously with similar ketogenic agents, except that we observed a longer duration to a higher peak *R*‐βHB (Crabtree et al., 2023; Stubbs et al., 2017) as a result of a larger amount (50 g) of BO‐BD consumed. It is likely we did not capture true peak *R*‐βHB before onset of exercise, as we did not observe any kinetic downtrend before maximal exercise. Although quantification with advanced imaging was not performed in this study, myocardial ketone uptake and utilization has been shown to positively correlate with plasma βHB concentrations (Gormsen et al., 2017; Nielsen et al., 2019). The reduction in glucose in both supplement conditions was expected due to the participants presenting to the facility while fasted along with the lack of carbohydrate in the beverages; the greater reduction in the BO‐BD condition has been observed before due to the modest glucose lowering effect of acute *R*‐βHB administration (Crabtree et al., 2023).

These two distinctly formulated beverages led to differing cardiopulmonary responses during the 120 min of rest before the maximal exercise test. BO‐BD consumption was associated with elevations in several measures of cardiopulmonary and ventilatory function, including HR, V̇E, V̇T, and V̇CO_2_. Exogenous ketosis has been observed to elicit acute elevations in cardiac function, including HR and cardiac output, in both healthy and cardiac patient populations (Nielsen et al., 2019; Selvaraj et al., 2022). We also observed increased oxygen consumption at rest between supplements. The resting ventilatory response to ketones observed here, elevated ventilation and tidal volume, may lead to enhanced lung perfusion, reduced physiological dead space, and improved alveolar gas exchange (Hallett et al., 2024).

While the specific mechanism responsible for this response is still not well understood, acute exogenous ketosis is known to produce an elevated cardiopulmonary state at rest (Berg‐Hansen et al., 2023, 2024; Dearlove et al., 2021; Gormsen et al., 2017; Nielsen et al., 2023), dramatically improving blood and oxygen availability due in part to an increased metabolic acidic load driving a hyper‐pulmonary response (Dearlove et al., 2019, 2021). Although speculative, this elevated pulmonary state as a consequence of acidic load potentially drives the downstream cardiovascular effects of ketones by promoting systemic afterload reduction, enhancing cardiac function. Supporting this, the pulmonary response is neutralized when ketones are consumed with bicarbonate to offset the acidic load (McCarthy et al., 2023). However, co‐ingestion with bicarbonate notably did not neutralize the elevated HR response (McCarthy et al., 2023).

We observed that resting cardiopulmonary differences maintained through submaximal stages of the exercise protocol but converged at high submaximal intensities. The only other study investigating exogenous ketosis exercise effects using the Bruce Protocol found no differences in performance in the first two stages, although this may be attributed to the use of ketone salts rather than BO‐BD, resulting in a different effect on acid–base status and comparatively lower peak *R*‐βHB (0.7 mM) (James & Kjerulf, 2019). Reduced exercise economy has been observed previously in individuals following a low‐carbohydrate, high‐fat diet who are in a state of nutritional ketosis (Burke et al., 2017) including submaximal exercise protocols combining exogenous ketones with very short‐term (5 day) KDs (Whitfield et al., 2021). It should be noted that a KD and exogenous ketones may be similar in achieving nutritional ketosis, but the metabolic and physiological responses are dramatically different in other aspects such as effects on insulin, lipolysis, and RER. Others, meanwhile, have found no difference in running economy following exogenous ketosis compared to placebo (Evans et al., 2019; James & Kjerulf, 2019). However, there is evidence elsewhere of differential running economy and lactate response following ketone supplementation, suggesting augmented oxygen consumption and altered exercise metabolism (Brady & Egan, 2024; Da Costa et al., 2020; Evans & Egan, 2018). In this study, we observed a complex exercise performance response that may explain the conflicting results from the literature, where cardiorespiratory differential response between supplements was augmented based on exercise intensity.

Despite both conditions producing highly similar total exercise durations, BO‐BD conferred consistently lower peak oxygen consumption (−6%) for completing the same workload within the same timeframe at maximal exercise intensity. However, there were no differences in any other cardiopulmonary measure between supplements in the maximal state. Notably, this discrepancy in oxygen consumption was only observed when oxygen demand was at its highest. It is important to note that the chosen maximal exercise protocol in this study, the Bruce Protocol, features standardized workloads at each stage, meaning participants reached peak exercise at the same workload on average regardless of supplement condition (Faull et al., 2019; Shaw, Merien, Braakhuis, Plews, et al., 2019). In a previous study of patients with Parkinson's disease, peak oxygen consumption following a supplementation protocol of exogenous ketones combined with carbohydrate was found to be similar to a solely carbohydrate regime (Norwitz et al., 2020); in that case, the study supplement likely created a state of competing metabolism and may not have shown the effects of ketone supplementation alone.

While speculative, our maximal oxygen consumption findings may hint at a potential energetic efficiency mechanism (Evans et al., 2017) that has been previously theorized generally (Cox & Clarke, 2014; Veech, 2004) and in the cardiovascular context of myocardial external efficiency (MEE), relevant to aerobic exercise performance (Ferrannini, Mark, & Mayoux, 2016). Ketone‐conferred increased metabolic efficiency has been observed in rats (Kashiwaya et al., 1997; Sato et al., 1995) and ex vivo in failing heart myocardial samples (Aubert et al., 2016). However, these results have not yet been translated to humans, where advanced imaging with PET observed similar efficiency at rest in both healthy adults and patients with HF compared to placebo (Nielsen et al., 2019), nor have they been observed in other similar preclinical investigations (Petrick et al., 2020). No physiological measures of MEE have been investigated during exercise in humans in nutritional ketosis and thus this remains a major question that should be addressed in future research.

Given all participants were adults who frequently exercise, and each served as their own control comparison that occurred within 1 week, it is unlikely the lower oxygen consumption at peak aerobic exercise was due to biomechanical differences that would decrease exercise economy. Ketone metabolism in the heart has inherently high heat of combustion per C_2_ unit (Veech, 2004), conferring more potential energy to the electron transport chain, thereby increasing redox span (Sato et al., 1995; Veech, 2004) and thus ATP generating efficiency (Cox & Clarke, 2014; Sato et al., 1995; Veech, 2004). In theory, this speculative effect reduces the cost of ATP production, promoting greater efficiency. If this effect is indeed manifested in humans during exercise, it is likely not driven by ketone oxidation in skeletal muscle, which is approximately 2%–5% of energy expenditure (Dearlove et al., 2021) accompanied by rapid saturation (Mikkelsen et al., 2015). Future acute ketone investigations must target exercise across the exercise intensity spectrum to better understand this effect and how it relates to performance.

In an acute exogenous state of ketosis, we observed cardiopulmonary response to differ based on physiological stress: initially elevated resulting in increased heart rate at rest or light exercise, with decreased oxygen consumption at peak workload, despite the achievement of the same workload. During higher intensities of exercise, there was a crossover point whereby the BO‐BD and PL conferred similar oxygen consumption. Ventilation, tidal volume, and V̇CO_2_ also highlight this potential crossover point near maximal intensity aerobic exercise. However, due to inter‐subject differences in maximal exercise capacity, we are unable to examine higher workload stages in detail with sufficient statistical power. As such, there is a need to conduct a similar supplementation exercise study in a more homogenously athletic cohort to further examine this seeming cardiopulmonary performance switch at higher exercise intensities.

While the results of this study inform the future clinical potential of these agents, the results must be interpreted within the context of the study population of healthy recreationally active adults. Additionally, we used a fat‐based beverage as a placebo that may have influenced results compared to water only or a carbohydrate‐based placebo, which may have influenced insulin and substrate oxidation, but likely not postprandial inflammatory responses, which are similar between high‐fat and high‐carbohydrate meals (Gregersen et al., 2012). The population of interest for potential future therapeutic ketone application is HF patients with both reduced exercise capacity and an underlying metabolic pathology resulting in reduced myocardial energetics that ketone metabolism could theoretically mitigate. However, it is unknown how elevated resting cardiac output would impact cardiac reserve and thus exercise capacity, both crucial to understand for safety in these patients. Here, we provide evidence that increased oxygen consumption at rest does not necessarily reduce cardiac reserve but instead preserves oxygen consumption at high levels of cardiac demand in healthy adults. A reduction in oxygen consumption at high relative percentages of maximal oxygen consumption, which can represent light to moderate bulk workloads in clinical populations, may help to correct the complex pathophysiological mismatch between ventilation and required oxygen undergirding dyspnea (Coccia et al., 2016), characteristic of HF. This effect of ketones at maximum oxygen consumption, in addition to its effect at increasing resting blood flow (Gormsen et al., 2017; Berg‐Hansen et al., 2023; Nielsen et al., 2019), lowering pulmonary pressure (Berg‐Hansen et al., 2024; Nielsen et al., 2023), and increasing ventilation, positions ketones as a potentially effective modulator of cardiopulmonary function across the physiological spectrum. Similar exercise studies should be reproduced with relevant clinical populations to better determine clinical usefulness and safety.

## 5 CONCLUSION

This study demonstrated that acute BO‐BD supplementation elevated resting cardiopulmonary function but reduced peak exercise oxygen consumption compared to a calorie and volume matched placebo in a healthy and active volunteer population. Further studies are needed to determine if these effects can benefit patients with impaired functional capacity caused by heart failure or other cardiopulmonary diseases.

## AUTHOR CONTRIBUTIONS

C.D.C., J.S., and J.V. conceived and designed research. C.D.C., J.S., A.B., B.R., D.D., A.C., X.ES., E.M., and A.J. performed experiments. C.D.C. and J.S. analyzed data. C.D.C., J.S., T.M., and J.V. interpreted results of experiments. C.D.C., J.S., and A.B. prepared figures. C.D.C. drafted manuscript. C.D.C., J.S., A.B., B.R., D.D., A.C., X.ES., E.M., A.J., T.B., M.K., T.M., Y.H., O.P.S., and J.V. edited and revised manuscript. C.D.C., J.S., A.B., B.R., D.D., A.C., X.ES., E.M., A.J., T.B., M.K., T.M., Y.H., O.P.S., and J.V. approved final version of manuscript.

## FUNDING INFORMATION

The study was supported in part by a Department of Defense grant W81XWH‐22‐1‐0867 awarded to the Ohio State University (Y.H., J.V., and O.P.S.). Study product and placebo were provided by Juvenescence.

## CONFLICT OF INTEREST STATEMENT

We have no disclosures to report.

## ETHICS STATEMENT

The study was conducted according to the guidelines of the Declaration of Helsinki, and approved by the Institutional Review Board (or Ethics Committee) of The Ohio State University (2022H0341; 12/12/2022). Informed consent was obtained from all subjects involved in the study.

## Data Availability

Data can be made available upon request to the corresponding author.
